# One new and seven newly recorded Callichromatini species from China (Coleoptera, Cerambycidae, Cerambycinae)

**DOI:** 10.3897/zookeys.275.4576

**Published:** 2013-03-04

**Authors:** Eduard Vives, Mei-ying Lin

**Affiliations:** 1 Key Laboratory of Zoological Systematics and Evolution, Institute of Zoology, Chinese Academy of Sciences, Beichen West Road, Chaoyang Dist., Beijing, 100101, China; 2 Museu de Ciuències Naturals de Barcelona, c/Sant Antoni, 73, 08221 Terrassa (Barcelona) Spain

**Keywords:** Callichromatini, new subgenus, new species, new records, China, Oriental region

## Abstract

One new species, *Schwarzerium yunnanum* **sp. n.** is described from Yunnan Province, China. And a new subgenus *Rugosochroma* **subgen. n.** is erected for it. Additionally, Seven species of the tribe Callichromatini are newly recorded from China: *Aphrodisium niisatoi* Vives & Bentanachs, 2007, *Aphrodisium tricoloripes* Pic, 1925, *Chelidonium violaceimembris* Gressitt & Rondon, 1970 (new from Vietnam too), *Chloridolum grossepunctatum* Gressitt & Rondon, 1970 (new from Vietnam too), *Chloridolum semipunctatum* Gressit & Rondon 1970, *Embrikstrandia vivesi* Bentanachs, 2005 and *Laosaphrodisium subplicatum* (Pic, 1937).

## Introduction

The recent visit of the first author to the IZAS Collection (Institute of Zoology, Chinese Academy of Sciences, Beijing), enabled the identification of many Callichromatini species along with interesting observations, some of them described in this work. The Callichromatini material in IZAS was not well studied before this work, with many specimens only identified at generic level. Seven species were found to be new for the Chinese fauna and herein reported for the first time. Meanwhile, one new subgenus and species are described from Yunnan.

### Specimens depository are abbreviated as follows in the description:

**BPBM** Bernice P. Bishop Museum, Honolulu, USA

**CCCC** Collection of Chang-chin Chen, Tianjin, China

**CJBB** Collection of Joan Bentanachs, Barcelona, Spain

**EVC** Eduard Vives collection, Terrassa, Spain

**IZAS** Institute of Zoology, Chinese Academy of Sciences, Beijing, China

**MNHN** Muséum National d’Histoire Naturelle, Paris, France

## Results

### Schwarzerium (Rugosochroma) subgen. n.

#### Type species.

*Schwarzerium (Rugosochroma) yunnanum* sp. n.

#### Description.

See “Diagnosis” below.

#### Etymology.

*Rugoso+chroma* in reference of this new subgenus have wrinkled all the pronotal and elytral surface. *Rugoso* meaning wrinkled in Latin and *chroma* meaning colour in Greek. Masculine gender.

### Schwarzerium (Rugosochroma) yunnanum sp. n.

urn:lsid:zoobank.org:act:EEEC8301-4F43-4F39-B9D5-226188FBEABC

http://species-id.net/wiki/Schwarzerium_yunnanum

Figs 1–2

#### Description.

Ground integument color bluish green, more intensely bluish and with long silvery pubescence underneath; antennae and legs bluish black; tarsi black, except slightly reddish onychium; head and pronotum shiny golden green; scutellum bluish green; elytra bluish green broadly along suture, with golden green dorsal longitudinal stripe, reaching from base to apex of elytra, cupreous golden sides from humeri to apex, and bluish epipleural margin.

Head large, transverse, strongly punctured, longitudinally furrowed from interantennal space to epistome; epistome short, straight, strongly punctured. Mandibles short, thick, slightly bent apically. Labrum trapezoidal, free, covered by fossulae and abundant golden setae. Eyes microfaceted, weakly protruding; upper lobe much smaller than lower. Antennae long, covered by long black setae and reaching apical 1/5 of elytra in males and apical quarter in females; segments saw-like beyond fifth antennomere, each segment (except first and second) with strong longitudinal outer margin demarcating two longitudinal porous areas.

Pronotum transverse (17:24), with strong transverse anterior depression on disc and two posterior transverse medially prominent humps; anterior border simple, posterior border weakly margined; sides armed with short median smooth bulge and smaller protuberance close to anterior angle; surface of pronotum strongly punctured, with golden setae at sides. Prosternum nearly smooth, shiny, with transverse striation at anterior half; prosternal process broad, punctured, expanded posteriorly to enclose procoxal cavities behind. Mesoventrite short, transverse, strongly punctured, wide between mesocoxae. Metaventrite longitudinally furrowed, finely punctured, covered by dense silvery white pubescence. Abdominal ventrites rather smooth and glossy, weakly punctured, sparsely pubescent; puncturation on pygidium stronger.

Scutellum triangular, margined laterally, smooth, depressed medially. Elytra long, narrow (11:4), sides subparallel; humeri round, protruding; suture fine, unmarginated; apex of elytra broadly round, with slightly marked sutural angle; surface of elytra rough, particularly at basal third, less so at apical quarter, covered by very sparse short, fine silvery tomentum; pubescence in apical area longer, denser and black.

Legs short and slender; profemora enlarged medially, meso- and metafemora widened apically; mesotibiae slightly arched, metatibiae flattened; pro- and mesotarsi short and wide; first metatarsomere laterally compressed, and remaining metatarsomeres short and broad.

**Figures 1–8.**
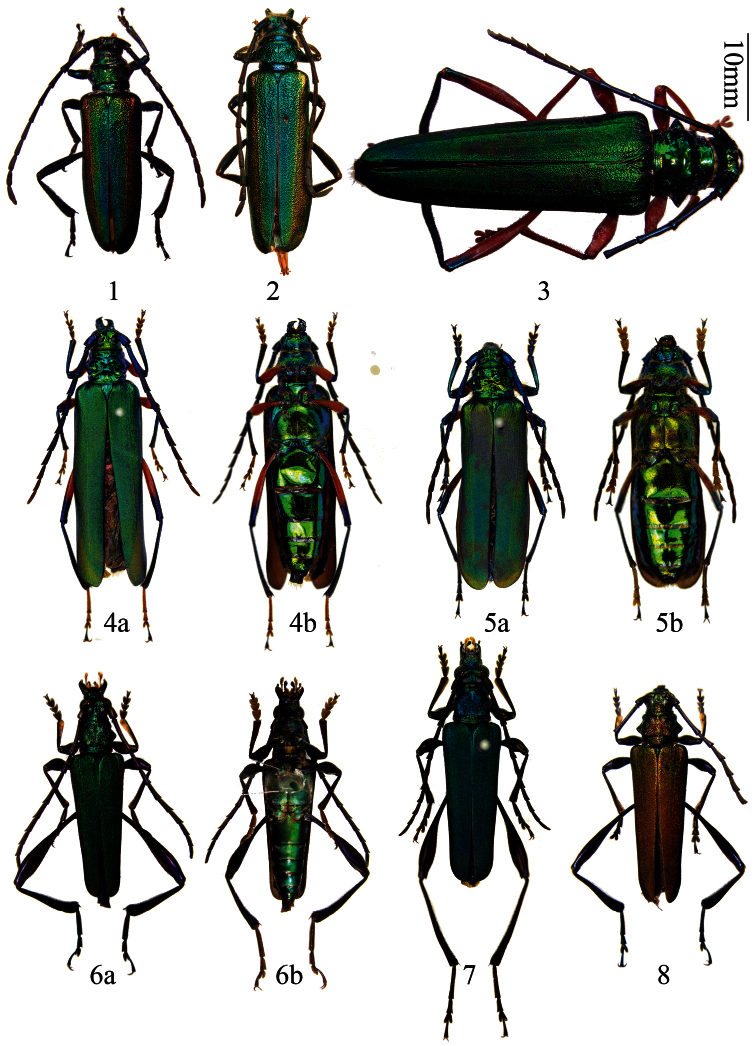
*Schwarzerium (Rugosochroma) yunnanum* subgen. n., sp. n., holotype male, from Yunnan **2** paratype, female, from Yunnan (in EVC) **3** *Aphrodisium niisatoi* Vives & Bentanachs, 2007, female, from Yunnan **4–5** *Aphrodisium tricoloripes* Pic, 1925 **4** female, from Guizhou **5** female, from Yunnan **6–8** *Chelidonium violaceimembris* Gressitt & Rondon, 1970 **6** male, from Hainan **7** female, from Hainan **8** female, from Yunnan. **a**. dorsal view. **b**. ventral view. Scale 10 mm.

#### Diagnosis.

This new species is similar to *Schwarzerium provosti* (Fairmaire) in its golden coloration and rough pronotum, but *Schwarzerium yunnanum* can be distinguished by the smaller size, elytra more parallel, and very short unicolor legs without club-shaped mesofemora. It differs from every other species in the genus *Schwarzerium* Plavilstshikov in the second metatarsomere not compressed. Based on the divergence in this character, together with the distinctive shape of mesofemora, we propose to establish a new subgenus, *Rugosochroma* subgen. n. (noun, masculine), with *Schwarzerium yunnanum* sp. n. as the subgeneric type.

#### Etymology.

The species is named after the type locality “Yunnan”.

#### Distribution.

China: Yunnan.

#### Specimens examined.

**China, Yunnan** **Prov.: holotype:** male, Yunnan, Zhongdian, Chongjianghe, alt. 1800 m, 1984.VIII.6, leg. Jianguo Fan (IZAS, IOZ(E) 1859281). **Paratypes:** 1 male (IZAS, IOZ(E) 1859283) and 1 female (EVC, ex IZAS, IOZ(E) 1905092), same data to holotype; 1 female, Yunnan, Lijiang, Yulongshan, alt. 2800 m, 1984.VIII.6, leg. Ruiqi Wang (IZAS, IOZ(E) 1859282).

### Aphrodisium niisatoi

Vives & Bentanachs, 2007

http://species-id.net/wiki/Aphrodisium_niisatoi

Fig. 3

Aphrodisium niisatoi Vives & Bentanachs, 2007: 635, figs 1 (holotype), 2–8.

#### Remarks.

This species is very typical with sexual dimorphism represented by larger mandibles in males than females.

#### Distribution.

China (**new country record**): Yunnan; Vietnam.

#### Specimens examined.

**China, Yunnan Prov.:** 1 female, 8 km North of Simao, 1957.V.22, leg. A. Monchadsky (IZAS); 1 male and 1 female, Yunnan, Mt. Kabi-ke, Menglian, 2006.VI.2, leg. local collector (EVC). **Vietnam, Vinh Phuc Prov.:** holotype, male, Tonkin, Mt. Tam Dao, 2001.VI. leg. Local collector (EVC).

### Aphrodisium tricoloripes

Pic, 1925

http://species-id.net/wiki/Aphrodisium_tricoloripes

Figs 4–5

Aphrodisium tricoloripes Pic, 1925: 18.
Aphrodisium (s. str.) *tricoloripes*; Podaný 1971: 270, 276.
Aphrodisium tricoloripes ; Breuning and Itzinger 1943: 39.

#### Remarks.

This is a very rare species only known from China, Myanmar and Vietnam. It is close to *Aphordisium cribricolle* Poll, 1890, but can be separated by the morphological feature of pronotum and the typical color of the legs.

#### Distribution.

China (**new country record**): Guizhou, Yunnan; Myanmar (Breuning and Itzinger 1943), Vietnam.

#### Specimens examined.

**China, Guizhou Prov.:** 1 female, Guizhou, Tongren, Jiangkou, Fanjingshan, 4500bu, alt. 1775, 2010.V-IX, leg. local collector (CCCC); **China, Yunnan Prov.:** 1 female, Yunnan, Deqin county, 28°28.835'N, 98°51.140'E–28°26.610'N, 98°55.212'E, alt. 3000–3500 m, 2006.VIII.10, light trap, leg. Xiaodong Yang (CCCC, 06B0683). Holotype, male, Tonkin, Anam. (MNHN, ex Collection M. Pic).

### Chelidonium violaceimembris

Gressitt & Rondon, 1970

http://species-id.net/wiki/Chelidonium_violaceimembris

Figs 6–8

Chelidonium violaceimembris Gressitt & Rondon, 1970: 151, fig. 26 d.

#### Remarks.

This is a typical oriental species. Very common in Laos and Vietnam and should be common in South China too.

#### Distribution.

China (**new country record**): Hainan, Yunnan; Laos, Vietnam (**new country record).**

#### Specimens examined.

**China, Yunnan Prov.:** 1 female, Yunnan, Xishuangbanna, Menghun, alt. 1200–1400 m, 1968.V.22, leg. Yiran Zhang (IZAS). **China, Hainan Prov.:** 2 males, Hainan, Baisha county, Yinggeling, alt. 600–780 m, 2011.IV.27–30, leg. Wenhsin Lin (IZAS & CCCC); 1 male, Baisha county, Yinggeling, Yinggezui, 2011.IV.30, leg. Yiting Chung (CCCC); 1 male 1 female, Wuzhishan, Dengshandao (entrance), 18.90840°N, 109.67359°E, alt. 708 m, 2010.IV.10, leg. Kuiyan Zhang (IZAS); 1 female, Hainan, Wuzhishan, 2010.IV.9, leg. WenI Chou (CCCC); 1 male, same data but 2010.IV.7; 1 female, Hainan, Qiongzhong county, Limushanzhufeng, 19.17863°N, 109.75071°E, alt. 840 m, 2010.IV.6, leg. Meiying Lin (IZAS); 3 males, Jianfengling, 2010.IV.13, leg. Wenhsin Lin (CCCC). **Vietnam, Vinh Phuc Prov.:** 3 males 2 females, Tam Dao National Park, 2011.VI.12 (EVC). **Laos, Vientiane Prov.:** holotype, male, Phou Khao Khoay, 1040 m, 1965.V.31, leg. J. A. Rondon (BPBM, examined by E. Vives in 2007.).

### Embrikstrandia vivesi

Bentanachs, 2005

http://species-id.net/wiki/Embrikstrandia_vivesi

Figs 9–12

Embrik-Strandia vivesi Bentanachs, 2005: 2, 3, figs 1 (holotype male), 2 (female), 3–4, 8–10.

#### Remarks.

This species is highly polymorphic in elytral and pronotal coloration. The IZAS collection contains specimens showing the base of elytra completely red, and the series from Yunnan includes specimens with their pronotum black and reddish.

#### Distribution.

China (**new country record**): Yunnan; Laos.

#### Specimens examined.

**China Yunnan Prov.:** 5 males 3 females, Yunnan, Jinping, Mengla, alt. 400 m, 1956.IV.28–29, leg. Keren Huang et. al (IZAS); 2 males, same data but 1956.IV.24; 1 male, same data but 1956.V.1; 1 male 1 female, same data but alt. 500 m, 1956.V.2; 1 male, Yunnan, Xishuangbanna, Xiaomengyang, alt. 850 m, 1957.VI.14, leg. Lingchao Zang (IZAS); 5 females, Yunnan, Xishuangbanna, Menghun, alt. 750 m, 1958.VI.2–7, leg. Xuwu Meng et al (IZAS); 1 female, same data but alt. 1200 m, 1958.V.31. **Laos, Xieng Khouang Prov.:** holotype, male, Laos, Xieng Khouang, 1997.VI (CJBB).

**Figures 9–12.**
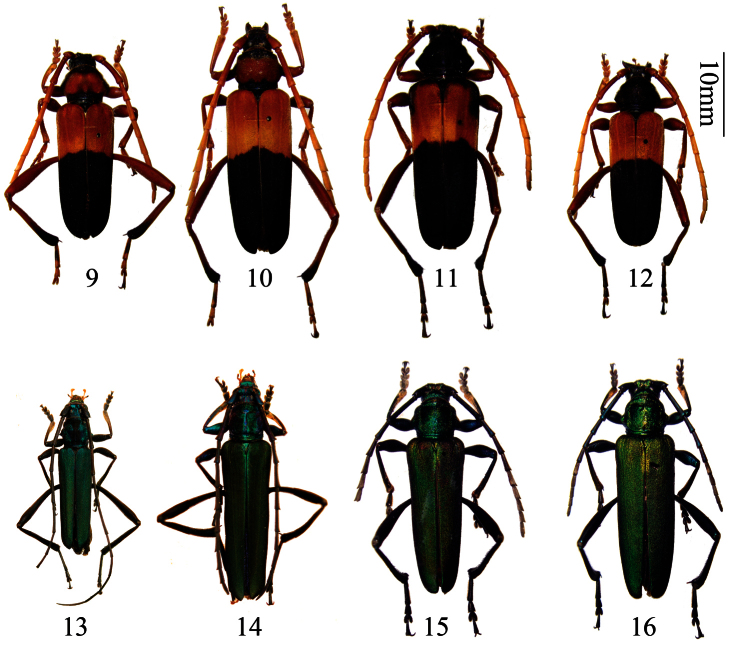
*Embrikstrandia vivesi* Bentanachs, 2005. **9** male, from Yunnan, pronotum mostly red **10** female, from Yunnan, pronotum mostly red **11** female, from Yunnan, pronotum mostly black **12** male, from Yunnan, pronotum mostly black **13** *Chloridolum grossepunctatum* Gressitt & Rondon, 1970. male, from Yunnan **14** *Chloridolum semipunctatum* Gressit & Rondon 1970. male, from Yunnan **15–16** *Laosaphrodisium subplicatum* (Pic, 1937) **15** male, from Guizhou **16** female, from Guizhou. Scale 10 mm.

### Chloridolum grossepunctatum

Gressitt & Rondon, 1970

http://species-id.net/wiki/Chloridolum_grossepunctatum

Fig. 13

Chloridolum (s. str.) *grossepunctatum* Gressit & Rondon 1970: 170, fig. 29a.

#### Remarks.

This is a small species described from Laos and it is very common in North Vietnam.

#### Distribution.

China (**new country record**): Yunnan; Laos; Vietnam **(new country record)**.

#### Specimens examined.

**China, Yunnan Prov.:** 2 males, Yunnan, Mt. Daningshan, 2012.VI.7, leg. local collector (EVC). **Vietnam, Vinh Phuc Prov.:** 2 males 3 females, N. Vietnam, Tam Dao National Park, 2011.VI.12, leg. E. Vives (EVC). **Laos, Vientiane Prov.:** holotype, male,Laos, Vientiane Prov., Ban Van Heua, 1035 m, 1965.IV.30 (BPBM, Bishop 8361, examined by E. Vives in 2007.).

### Chloridolum semipunctatum

Gressit & Rondon 1970

http://species-id.net/wiki/Chloridolum_semipunctatum

Fig. 14

Chloridolum (s. str.) *semipunctatum* Gressit & Rondon 1970: 171, fig. 29b.

#### Remarks.

This is a very rare species in Northern Laos. The morphology of this species is very different of other *Chloridolum* species.

#### Distribution.

China (**new country record**): Yunnan; Laos.

#### Specimens examined. 

**China, Yunnan Prov.:** 1 male, Mt. Gaoligongshan, 2012.VI.12, leg. local collector (EVC). **Laos, Sayaboury Prov.:** holotype, male, Laos, Sayaboury (Xaignabouri), 280 m, 1966.V.20 (BPBM, Bishop 8362, examined by E. Vives in 2007.).

### Laosaphrodisium subplicatum

(Pic, 1937)

http://species-id.net/wiki/Laosaphrodisium_subplicatum

Figs 15–16

Chelidonium gibbicolle v. *subplicatum* Pic, 1937: 11.
Aphrodisium (s. str.) *subplicatum*; Gressitt and Rondon 1970: 149, fig. 25 i.
Chelidonium subplicatum ; Podaný 1974: 7, 42.
Laosaphrodisium subplicatum ; Bentanachs 2012: 81.

#### Remarks.

This species was originally described as a variety of *Chelidonium gibbicolle* by Pic (1937). Gressitt and Rondon (1970) treated it as a species and combined it to the genus *Aphrodisium*. However, it was transferred to the genus *Laosaphrodisium* Bentanachs (2012) based on the following characters: body dull green, without yellow bands, discal area of pronotum with two longitudinal stripes of black pubescence.

Although Bentanachs (2012) wrote “China (Yunnan, Gressitt 1950)” under the distribution of this species, it was a big mistake (personal communication with Bentanachs in Dec. 2012). Gressitt did not report any record of *subplicatum* from China and Bentanachs did not examined any specimens from Yunnan. The reliable locality from China is only Guizhou up to now. Yunnan is a possible locality but it need the confirmation of specimens.

#### Distribution.

China (**new country record**): Guizhou; Laos, Vietnam.

#### Specimens examined.

**China, Guizhou Prov.:** 1 female, Guizhou, Shiqian, Jinxing, alt. 670 m, 1988.VII.24, leg. Shuyong Wang (IZAS); 1 female, same data but alt. 670–800 m; 1 male, Guizhou, Shiqian, Jinxing, alt. 800 m, 1988.VII.25, leg. Hongxing Li (IZAS). **Vietnam, Vinh Phuc Prov.:** 2 males 1 female, Vinh Phuc Prov., Tam Dao National Park, 2011.VI.20, leg. E. Vives (EVC); holotype, male, Vietnam, Tonkin, Hoa-Binh (MNHN, ex Collection M. Pic).

## Supplementary Material

XML Treatment for Schwarzerium (Rugosochroma)

XML Treatment for Schwarzerium (Rugosochroma) yunnanum

XML Treatment for Aphrodisium niisatoi

XML Treatment for Aphrodisium tricoloripes

XML Treatment for Chelidonium violaceimembris

XML Treatment for Embrikstrandia vivesi

XML Treatment for Chloridolum grossepunctatum

XML Treatment for Chloridolum semipunctatum

XML Treatment for Laosaphrodisium subplicatum
